# Endogenic heat at Enceladus’ north pole

**DOI:** 10.1126/sciadv.adx4338

**Published:** 2025-11-07

**Authors:** Georgina Miles, Carly J. A. Howett, Francis Nimmo, Douglas J. Hemingway

**Affiliations:** ^1^ Southwest Research Institute, Boulder, CO, USA.; ^2^ Department of Atmospheric, Oceanic and Planetary Physics, University of Oxford, Oxford, UK.; ^3^ Planetary Science Institute, Tucson, AZ, USA.; ^4^ Department of Earth and Planetary Sciences, University of California, Santa Cruz, CA, USA.; ^5^ University of Texas at Austin, Austin, TX, USA.

## Abstract

The long-term survival of Enceladus’ ocean depends on the balance between heat production and heat loss. To date, the only place where a direct measurement of Enceladus’s heat loss has been made is at the south pole. Here, we show that the north pole also emits heat at a greater rate than can be explained by purely passive models. By comparing winter and summer observations taken with the Cassini Composite InfraRed Spectrometer, we find a winter temperature ~7 kelvin warmer than passive modeling predicts, accounting for uncertainties in emissivity and thermal inertia. An additional endogenic heat flux of 46 ± 4 milliwatts per square meter is required to match the observed radiance. The implied local shell thickness is 20 to 23 kilometers—consistent with the higher end of thickness models based on gravity, topography, and libration measurements. This work provides a previously unidentified constraint for models of tidal heat production, shell thickness, and the long-term evolution of Enceladus’ ocean.

## Teaser

Unique observational evidence of heat from the warm ocean conducted through the water ice shell is found at Enceladus’ north pole.

## INTRODUCTION

The energy budget of Enceladus is an important quantity to evaluate because its tidal heat, generated from its interaction with Saturn via the orbital resonance with Dione, is linked to Enceladus’ age, ice shell thickness, and thus, the lifetime of its ocean (*1*). A long-lived stable ocean is necessary for life to evolve (*2*), and yet we do not know if Enceladus has met that condition as the ages of all of Saturn’s satellites are still uncertain, ranging from <2 to 4.5 billion years (*3*–*5*). This is further complicated by uncertainty in both the bombardment history in the Saturn system (*6*) and, on Enceladus in particular, its long-term orbital evolution (*1*, *7*).

Enceladus has a cold and bright surface, atop a predominantly water ice shell above a global ocean, decoupled from its inner rocky core (*8*). The ice shell is the thinnest at the poles (*9*, *10*), and at the surface of the ice shell, its temperature is governed by its albedo and thermal inertia. Enceladus has the highest bolometric albedo of any of Saturn’s satellites, ranging from 0.74 to 0.96 (*11*–*13*). While parts of Enceladus are young and show evidence of resurfacing events (*14*), it is by no means uniform. Surface thermal inertia varies zonally and, including uncertainty because of the limitations of observations, ranges from 3 to 50 J m^−2^ K^−1^ s^−1/2^ (MKS units), with a global average of 15 MKS (*11*).

Cryovolcanic activity in Enceladus’ South Polar Terrain (SPT) was observed by the Cassini mission instruments, where frozen particles and vapor erupt from long and narrow fissures dubbed sulci or Tiger Stripes (*15*, *16*). The surface temperatures in the SPT (120 K and above) are elevated compared to its surroundings (60 to 80 K) and are particularly enhanced within and between the Tiger Stripes (*17*–*19*). The endogenic power is so substantial that it can be measured by inferring the observed excess heat compared to that expected from passive models and is found to be between 4 and 19 GW, with the uncertainty being due to studies using varying assumptions of SPT thermophysical properties and wavelength ranges (and, thus, temperature sensitivity) (*18*, *19*). Conductive heat loss is expected throughout the ice shell, but higher shell thicknesses result in lower conductive heat fluxes, so detection of endogenic anomalies outside the SPT is challenging.

The thickness of Enceladus’ ice shell has been estimated by geodetic observations from Cassini (*10*, *20*–*23*), but interpretation of the gravity, topographic, and libration data is not straightforward. As a result, independent constraints, such as the local heat flux estimate that we provide below, can help distinguish between different models.

A shell thickness model was used in (*10*) to derive a global conductive heat loss of 20 to 35 GW (not including that emitted by the Tiger Stripes or latent heat released in the plumes themselves). References (*21*, *23*) estimate global values between 25 and 30 GW and 18 and 28 GW, respectively, with the latter estimate deriving from a larger mean ice shell thickness (27 to 33 km). In terms of heating, a potential range of equilibrium tidal heating rates (1.8 to 150 GW) is suggested in (*5*, *24*) depending on (among other things) whether a torque on Dione is or is not present, and ref. (*25*) suggests a present-day value of ~50 GW. How long the global ocean can persist is directly related to whether heat loss rates can be balanced with heating rates over time.

Evidence of conductive heat flow or thermally anomalous hotspots is more likely to be found under two circumstances: where the ice is the thinnest and at night/during winter when temperatures are the coldest. Because of the temperature T^4^ term in the Stefan-Boltzmann relationship between power and temperature, a unit change in power produces a larger change in temperature at cold temperatures than in warm scenes. To illustrate, for unit emissivity, an addition in available power of 50 mW m^−2^ at 30 K results in a 6 K temperature increase, while at 60 K, it would result in an apparent 1 K increase. To identify an area as warmer than expected from passive emission alone using thermal remote sensing, such changes would need to exceed the observed temperature uncertainty (which increases with decreasing temperature) and estimated model uncertainty, which depends primarily on the thermal inertia, albedo, and emissivity (see below).

We identify two sets of observations from the Cassini Composite InfraRed Spectrometer (CIRS) (*26*) in north polar winter and summer that are best explained by endogenic heat contributing to the observed radiance. Alternative explanations are explored but do not satisfy both observations nor are they likely given what is independently understood about the thermal properties of the near-surface ice at the north pole of Enceladus. The quantity of endogenic heat and the ice shell thickness it implies are broadly consistent with current model estimates using gravity/libration data and may be able to distinguish between competing models.

## RESULTS

### Cassini CIRS winter north polar temperature results

**Fig. 1.**
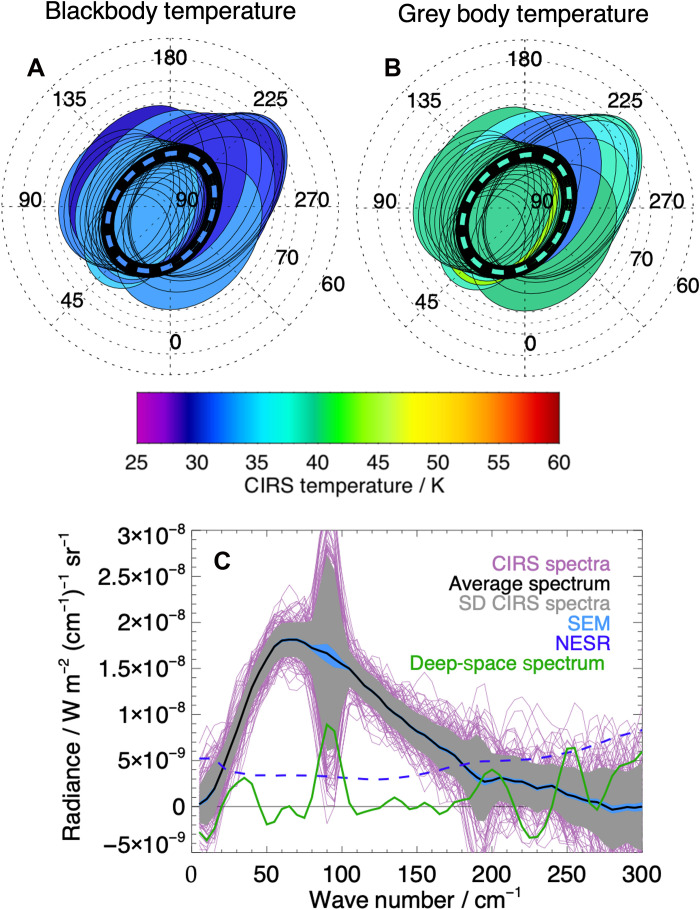
CIRS FP1 observations of Enceladus’ north pole. CIRS FP1 FOV in the northern polar region observed on 14 July 2005 during northern hemisphere winter. The pole is in winter darkness. Temperatures correspond to (**A**) a single temperature blackbody fit and (**B**) (improved) gray body temperature joint fit with emissivity to the individual spectra, with the location of the average stare FOV shown by the dashed line. The Saturn-facing hemisphere is centered at 0°W at the bottom of the panels. (**C**) Spectra of 110 stare observations corresponding to those shown in (A) and (B) over the north pole of Enceladus. Also shown are the averaged spectrum, the standard deviation of the stare spectra, and the standard error on the mean (SEM). In addition, the noise equivalent spectral radiance (NESR) appropriate for the original resolution of the interferogram (5 cm^−1^) is shown (blue dashed line). The nearest deep-space spectrum is shown in green to convey the minimum systematic and random noise of the instrument. The features around 90 and 190 cm^−1^ are well-documented artifacts of the CIRS FP1 spectra (*26*).

Figure 1 (A and B) shows temperatures derived from a stare observation of Enceladus’ north pole (location shown in Fig. 1), made during northern hemisphere winter on 14 July 2005 by CIRS Focal Plane 1 (FP1) (see Materials and Methods).

The individual spectra and stare are shown in Fig. 1C, along with the standard deviation and the standard error of the mean. It is apparent that for such cold scenes, individual spectra can be noisy, particularly above 200 cm^−1^, but their average provides a smoother spectrum to be fit. Also shown are the noise equivalent spectral radiance (noise floor) for FP1 at the spectral resolution of the measurement and the closest-in-time deep-space spectrum available to indicate the precision of an individual measurement.

At the time of the observation in 2005, Enceladus’ north pole had been radiatively cooling in polar winter darkness for 10 (Earth) years and exhibited extremely low (down to ~30 K) surface temperatures (Fig. 1). Figure 1 (A and B) shows both blackbody and nonblackbody (gray body) temperatures derived from the CIRS stare as well as other individual observations of the high northern latitudes from this encounter. We consider both blackbody and equivalent gray body temperature fits to the spectra here as many of these cold scenes were found to have unexpectedly low effective emissivities—i.e., the radiating efficiency with reference to a surface that is a blackbody (see below). In these cases, gray body fits follow the shape of the observation more closely.

**Fig. 2.**
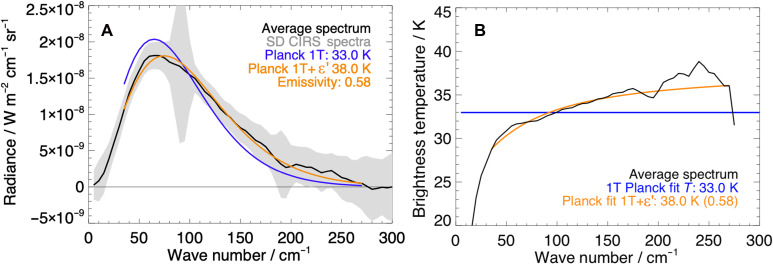
CIRS observation and temperature fit. (**A**) CIRS north pole radiance spectra from 14 July 2005 (gray), averaged spectrum (black), single temperature (1T) Planck function temperature fit (blue), and gray body temperature and effective emissivity (T+ ε′) fit (orange). (**B**) Averaged CIRS spectrum in BT.

It is generally sufficient to fit Cassini CIRS observations for Saturn’s icy moons with a single blackbody temperature Planck function as the effective emissivity is usually close to 1 (*26*, *27*) or when the origins of nonunit emissivity are unknown or highly uncertain, which can be the case for cold scenes with noisy spectra. Using the fitting method detailed in Materials and Methods, a conventional single temperature (1T) with a unit emissivity of $33.0_{-0.5}^{+0.5}$ K is fit to the averaged spectrum (Fig. 1C). However, in this case, a 1T fit (Fig. 2A in blue) does not reproduce the shape of the observed spectrum. At lower wave numbers up to and just before the peak, the fitted spectrum overestimates the radiance, and at higher wave numbers, it understates the tail. Furthermore, as Fig. 2B shows, the CIRS stare expressed as a brightness temperature (BT) spectrum is inadequately explained by a single blackbody temperature fit (a flat BT spectrum). The spectrum has a pronounced gradient and curvature. Gradients in BT with wave number are indicative of either a surface temperature gradient within the scene or nonunit emissivity (or, similarly, a variation in surface material/spectral emissivity).

Jointly fitting a single (gray body) temperature and effective (broadband) emissivity ε′ (labeled T+ ε′) yields a substantially improved fit (both in wave number and BT), particularly around the peak. Effective emissivity describes the surface’s radiative efficiency. A gray body temperature of 38 ± 0.5 K and an effective emissivity of 0.58 ± 0.03 (Fig. 2) are determined as a better fit to the observation. An effective emissivity of ~0.6 is considered very low for water ice at these wave numbers (*27*), and its potential origin and importance are discussed in the following section.

Spectra can be fit in other ways to represent the field-of-view (FOV) emission. A free fit of two blackbody temperatures ( $20.3_{-1.7}^{+2.4}$ and $39.6_{-0.49}^{+1.5}$ K) and their fractions of the FOV (0.54 and 0.46) produces a shape similar to the T+ ε′ (gray body) fit at most wave numbers but does not reproduce the curvature of the BT spectrum at wave numbers <50 cm^−1^ [a known feature of icy surfaces (*28*)]. We consider a surface temperature of ~20 K unphysically cold for Enceladus. Fixing a lower “passive” blackbody temperature and performing a free fit of a second, higher temperature and weighting produce small residuals but have no combination of two blackbody temperatures that has a root mean square difference quantitatively better than any other. There is currently no independent observational evidence than can break this degeneracy. All combinations result in similar total radiant power to the gray body fit but do not reproduce the falloff at low wave numbers (<60 cm^−1^) seen in the observed BT. We favor the gray body result because it fits the data better while giving a comparable but conservative heat flow estimate with no prior information.

### Enceladus’ emissivity

**Fig. 3.**
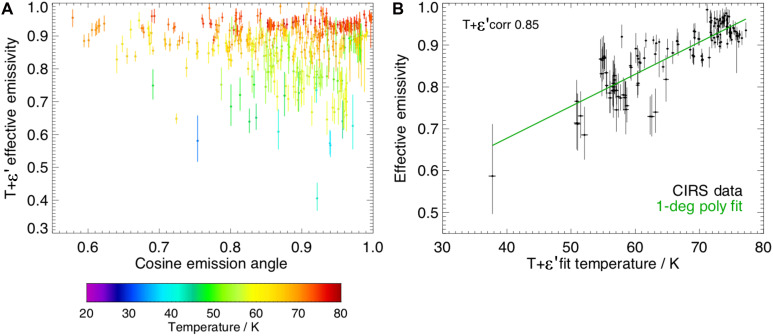
Emissivity and temperature relationship. (**A**) CIRS FP1 quasistares derived from all available observations with criteria area <10^5^ km^2^, FOV center latitude north of 50°S, and minimum number of contributing spectra of 10. The population is independent of the north polar stare observation. (**B**) Subset of (A) where the maximum area considered is 10^4^ km^2^ (except at high northern latitudes), where ε′ from the joint fit is plotted against blackbody temperature. The green line is a fit to the data, the equation for which is given in Eq. 1 (Materials and Methods).

The surfaces of the icy moons of Saturn have a high emissivity [<0.9 (*27*, *29*)], but the Enceladus north polar stare observation yields an effective emissivity considerably lower than this (0.58). We created a global study of ε′ derived from CIRS FP1 observations outside the SPT (Fig. 3A) to investigate how it varied with temperature and emission angle (see Materials and Methods).

Figure 3A shows the relationship between ε′ and cosine of emission angle for FOVs with area <10^5^ km^2^. The emissivity is broadly flat relative to the cosine of emission angle up to ~50° (few observations are made above this angle), making geometric effects unlikely to be responsible, although there is a strong dependency on temperature indicated by the color value, resulting in a “rainbow” effect. These data are refined further in Fig. 3B, where between 50°S and 60°N, the FOV areas are maximum 10^4^ km^2^ (~1% of Enceladus’ surface) to reduce the influence of spatial inhomogeneity and 10^5^ km^2^ above 60° (because of data paucity). Fit temperature and emissivity are highly correlated (0.85), with the downward trend of emissivity with temperature more pronounced for the coldest temperatures, although these often have higher fit uncertainty conferred by the lower signal-to-noise ratio of the observations. We use the empirical fit expressed in Eq. 1 (Materials and Methods) and shown in Fig. 3B in the surface thermal model (see the following section) to more accurately represent how efficiently the surface radiates throughout the Saturn year and to avoid overestimating any endogenic power that might be present. We illustrate how this relationship might arise, including from spectral emissivity effects, in our Discussion.

### Passive model results

We first estimate a baseline temperature using a passive surface thermal model (see Materials and Methods) to establish whether a 38 K gray body temperature is a higher temperature than would arise from purely passive emission (reradiated from solar heating).

**Fig. 4.**
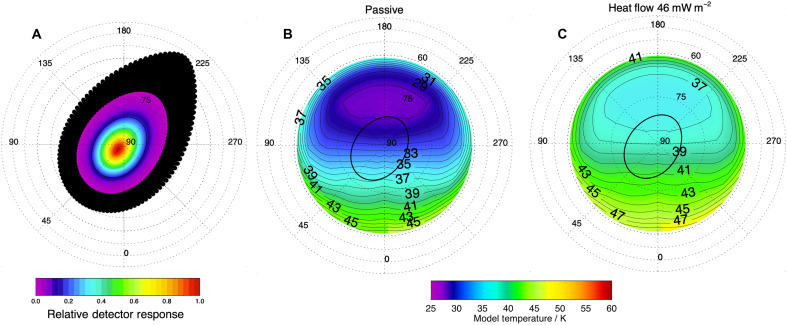
Projected detector response and modeled surface gray body temperature at the time of observation. The average CIRS FP1 FOV ellipse is shown in black. The model uses a bolometric albedo of 0.76, a thermal inertia of 16 MKS, and empirically derived ε′ for the model temperature (see Materials and Methods). The FOV is in winter polar darkness. The thermal gradient across the pole arises from infrared Saturn shine on the Saturn-facing hemisphere. (**A**) Projected FP1 detector response. (**B**) Passive model. (**C**) With an additional 46 mW m^−2^ endogenic heat input at the base slab of the model. (B) and (C) may be compared with Fig 1B.

Figure 4B shows the resulting modeled surface temperature at a sub-FOV resolution coincident with the observation, with the CIRS FP1 FOV overlaid. The longitudinal/hemispheric thermal gradient in the polar region apparent in Fig. 4B arises from the Saturn shine (reflected solar and thermal emission from Saturn). At Enceladus’ orbit, this is sufficient to affect the surface temperature during winter. The net effect is a variation in the modeled passive temperature within the CIRS scene of ~3 K (see also the discussion of roughness below and in Materials and Methods). Inverting for BT produces an almost flat spectrum (similar to the CIRS 1T fit), so passive temperature gradients within the scene are insufficient on their own to explain the observed BT gradient.

**Fig. 5.**
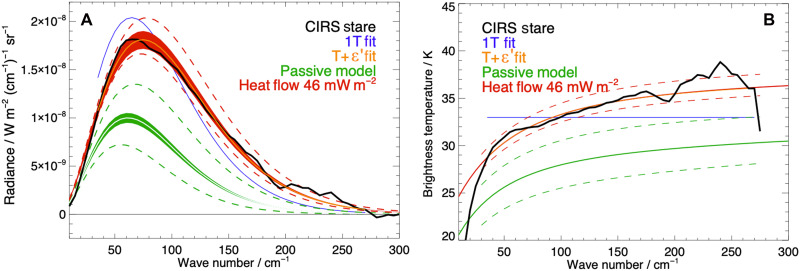
Modeled spectrum. (**A**) Observed, fit, and modeled CIRS spectra with modeled passive (green) and passive and endogenic (red) filled ranges of uncertainty arising from the error associated with the fitted CIRS effective emissivity. Dashed lines represent model uncertainty from the ranges of thermal inertia (3 to 33 MKS) and albedo (0.7 to 0.82). 1T and T+ ε′ fits are as defined for Fig. 2. (**B**) The same radiances are converted to BT.

Figure 5A shows the resultant modeled spectrum of passive emission (weighted by the FP1 detector response; Fig. 4A and Materials and Methods), representing how the modeled surface would be observed by CIRS FP1. Modeling the low ε′ for the scene produces curvature in BT with the same tendency as the observation but at a considerably lower temperature (Fig. 5B). This temperature would be lower if effective emissivity had not been accounted for in the seasonal model. As the observation occurs in deep polar night, the model uncertainty (shown by the dashed lines) is dominated by the thermal inertia [ $16_{-13}^{+17}$ MKS (*11*)], and the CIRS-derived albedo [0.76 ± 0.06 (*11*)] is secondary. These thermal inertia and albedo ranges were derived from a chi-squared minimization of model-observation difference using global CIRS surface temperatures made over several local times. The model used a wide range of thermal inertia and albedos, and the resultant range represents the model values that achieved global agreement within the observation error. Similar albedo ranges have additionally been derived independently from CIRS using other observation types (see Materials and Methods for further details).

This forward-modeled spectrum is fit, finding a passive gray body temperature of $31.2_{-3.1}^{+3.2}$ K, ~7 K colder than the joint fit to the CIRS observation. The temperature errors come principally from the uncertainty in albedo and thermal inertia. The latter dominates the uncertainty being six times larger than the CIRS fitting error. These passive results (Fig. 5A, green) fall far short of the observed radiance. The fit error in ε′ is represented by the filled green region of uncertainty in Fig. 5A.

### Endogenic heating

Figures 4C and 5 (in red) illustrate the effect of adding an endogenic heat source to the bottom layer of the thermal model. By iteration, a scene-wide base input of 46 mW m^−2^ produces a surface radiance that exactly matches the gray body fit to the observed spectrum for the expected values of thermal inertia and albedo (i.e., a gray body temperature of 38 ± 1 K). If we also consider the boundary cases of the albedo and thermal inertia uncertainty ranges (dashed lines in Fig. 5), then the equivalent range can be replicated by heat flows between 42 and 50 mW m^−2^.

To put this north polar result into context, the total endogenic emission in the SPT is estimated to be between 4 and 19 GW (*18*). If the endogenic power for the SPT is isotropically averaged below −65° latitude, it equates to 100 to 500 mW m^−2^. Thus, conductive heat flow at the north pole over the CIRS FOV of 42 to 50 mW m^−2^ is 8 to 50% of the endogenic emission averaged over the SPT. A flux of 46 mW m^−2^ is equivalent to 1.7 GW over the pole to 65°N (the area of SPT). The north polar region lacks the high concentration of emitted power that makes the SPT emission so readily apparent in thermal observations; the difference may also explain why the north polar region appears mostly old and undeformed while containing a few apparently relaxed impact craters. Our best-fit heat flux is marginally too small to explain the observed past relaxation of the impact crater Aladdin (*30*), which is situated at the lower latitude of 60°N; this hints at a higher (potentially transient) heat flux and thinner north polar ice shell in the past.

### Evaluation of endogenic heat scenario

While conductive heat loss is expected across Enceladus, and the addition of endogenic heat fits the observed radiance at the north pole, it is not the only possible explanation for higher-than-expected temperatures at the north pole. An unanticipated thermal inertia anomaly could act to slow winter heat loss. We investigated the possibility of such an anomaly either at or beneath the surface. A thermal inertia anomaly could occur at an unseen depth that is sufficient to affect the seasonal thermal wave but not the diurnal wave [the diurnal wave has been used to characterize surface thermal inertia and albedo with thermal observations; see (*11*)].

Our initial inspection showed that the surface thermal model requires a thermal inertia of 75 MKS to reproduce the observed radiance of the winter north polar stare passively [compare the expected global range of 3 to 50 MKS (*11*)]. A value this high is not seen elsewhere on either Enceladus or any of Saturn’s other icy moons (*11*, *31*, *32*). To evaluate the likelihood of such a thermal inertia anomaly, we compare the results of using model parameters that fit the observations in one season with those from another to discern the properties of the seasonal thermal inertia indirectly.

**Fig. 6.**
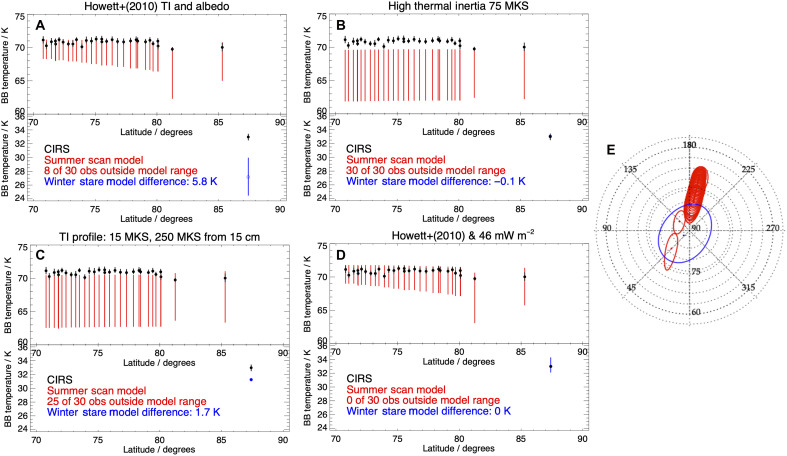
Observed and modeled summer (upper box/red) and winter (bottom box/blue) north polar temperatures. (**A**) Passive model of temperatures predicted by the range of thermal inertia and bolometric albedo derived in (*11*): $16_{-13}^{+17}$ MKS and 0.76 ± 0.06, respectively (red and blue), compared to the CIRS data (black). This model fails to match the winter stare observation (average location in blue). BB, blackbody. (**B**) High surface thermal inertia (75 MKS) case required to match the observed winter polar radiance. This model fails to match the summer/lower-latitude observations. (**C**) High subsurface thermal inertia (15 MKS at the surface and 250 MKS from 15-cm deep) profile that achieves a close match to summer observations but fails to match the winter stare. For (C) and (D), error bars in red result from bolometric albedo uncertainty as thermal inertia is fixed. (**D**) As (A) but with 46 mW m^−2^ endogenic heat added to the base of the model, which fits all the observations. (**E**) CIRS FP1 FOV in northern hemisphere summer (red) and the winter stare observation (blue).

In Fig. 6, we present four scenarios where CIRS observations in polar summer and winter are modeled using different thermal inertia or endogenic heat. We compare results for the winter polar stare and those from a polar scan with a smaller FOV observed on 14 October 2015 (see Materials and Methods), by which time the polar region was out of polar winter and had a sunlit diurnal cycle. These cases comprise the only other set of CIRS FP1 observations made across the north pole that are confined to the highest northern latitudes. Details of the summer polar spectra are given in Materials and Methods, and the spectra and Planck function fits to them are shown in the Supplementary Materials.

The model ranges, indicated in the panels of Fig. 6 by vertical lines, convey the limits of uncertainty in thermal inertia and albedo. The expected (*11*) thermal inertia and albedo passive model produces a temperature too cold in winter (as discussed above) but reproduces some CIRS temperatures in the summer polar scan (Fig. 6A). The high thermal inertia case, which exactly reproduces the winter stare temperature, produces summer temperatures that are all colder than the observations (Fig. 6B). In this case, the error bars result from uncertainty in the bolometric albedo as thermal inertia is fixed. The thermal inertia anomaly at the depth scenario (described in the caption) that most closely reproduces summer temperatures (although only matching 5 of 30 observations) produces a winter surface temperature that is too low and would require an additional component of endogenic heat to match the observation (Fig. 6C). The endogenic heat scenario (this work) that matches the winter CIRS observation exactly produces summer temperatures that coincide with all the CIRS observations (Fig. 6D). We conclude that endogenic heat is the most likely explanation for the excess in observed radiance at the north pole of Enceladus in 2005.

### Topographic roughness

The individual spectra comprising the summer polar observations (Fig. 6), together with phase and emission angle, are given in the Supplementary Materials. The spectra show that despite some mid-high emission angles, all spectra are well fit by blackbody Planck functions. Roughness effects would manifest as spectra that are harder to fit with increasing emission angle—an effect that is not observed (figs. S1 to S4).

Surface facets may cause sub-FOV temperature variation that may contribute to emissivity variation. However, there is precedent for expecting these to be minimal at Enceladus’ winter north pole. The winter pole of Enceladus’ neighbor, Rhea, was demonstrated in (*33*) to have limited thermal variation because of topography alone (<1 K)—despite being heavily cratered.

To extend this to Enceladus, we modeled the range of surface temperature inhomogeneity within its north pole stare FOV produced by facets up to 50° slope and a range of azimuth angles (see Materials and Methods). The model produced temperature differences of <0.2 K from when the surface was modeled as flat. As the FOV has no insolation, this is the incremental impact of Saturn shine on an elevated facet. The results suggest that temperature variations would not be changed appreciably by the presence of elevated facets.

### Ice shell thickness

**Fig. 7.**
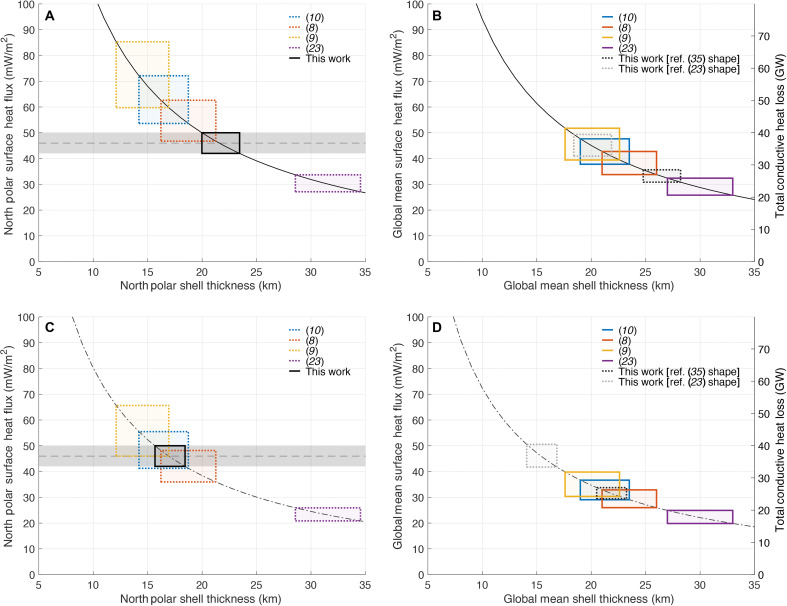
Ice shell thickness and conductive heat flux. Ice shell thickness and conductive heat flux are shown for the north polar region (**A** and **C**) and for the global mean (**B** and **D**). We consider both zero porosity ice [solid curves in (A) and (B)] and ice with a porous layer [dash-dotted curves in (C) and (D)] and, therefore, lower thermal conductivity (Materials and Methods). The curves assume surface temperatures of *T*_s_ = 51 K (A and C) or *T*_s_ = 60 K (B and D). Basal temperatures are *T*_b_ = 270 K in all cases. Solid rectangles in (B) and (D) show global mean shell thicknesses estimated from various published studies and the corresponding global mean surface heat flux. These shell thicknesses are extrapolated to the north polar region (Materials and Methods) and shown in (A) and (C) as dotted rectangles. Our estimated north polar heat flux is shown by the dashed horizontal line and shaded band in (A) and (C) with the solid black rectangle showing how this relates to the north polar shell thickness. This is extrapolated to the global mean thickness and shown as dotted rectangles in (B) and (D) using two different shape models (see Materials and Methods). Shell thickness uncertainties for the model are artificial (see Materials and Methods).

Given a surface heat flux, the conductive ice shell thickness may be inferred, assuming no internal heat sources [e.g., (*10*)]. Our estimated heat flux of 46 ± 4 mW m^−2^ implies a local north polar shell thickness of 20 to 23 km (Materials and Methods), which may be compared with other estimates in Fig. 7 that were derived independently from interior structure models on the basis of shape, gravity, and libration data. Our value (solid black rectangle in Fig. 7A) implies a thicker ice shell at the north pole than estimates in (*9*, *10*) and is marginally consistent with the estimate in (*8*) and thinner than the estimate in (*23*) (Fig. 7A). Equivalently, we can extrapolate our north polar result to the global mean (Materials and Methods). When we adopt the shape model in (*34*), the global mean thickness is ~5 km thicker than the north polar shell thickness, so our inferred global mean thickness is 25 to 28 km (black dotted rectangle in Fig. 7B). However, when we adopt the shape model in (*23*), the global mean is ~1 km thinner than the north polar thickness, so our inferred global mean thickness becomes ~18 to 22 km (dotted gray rectangle in Fig. 7B). This is because the shape model in (*23*) shows a topographic high at the north pole, whereas the shape models in (*34*) and others [e.g., (*35*–*37*)] exhibit a topographic low at the north pole (Materials and Methods). Although adopting the shape model in (*23*) leads us to extrapolate our result to a global mean thickness that is closer to the values estimated in (*8*–*10*), those models all assume a shape [either (*34*) or (*35*)] that is not consistent with the shape in (*23*), so this does not constitute an internally consistent solution. Likewise, extrapolating the global mean thickness in (*23*) (solid purple rectangle in Fig. 7B) to the north polar thickness (dashed purple rectangle in Fig. 7A) yields a result that is not consistent with our estimate for the north polar shell thickness.

Inclusion of ice shell internal heating would result in a thicker ice shell for the same surface heat flux. Conversely, a near-surface porous low-conductivity region would result in a thinner ice shell. In a region with long-lived amorphous ice (*38*) in the very cold temperature regime of the north pole, the transition from high-density to low-density amorphous ice can cause large cracks (i.e., porosity), which might have implications for how heat is transferred to the surface (*34*, *38*). Adopting values for the thickness and porosity of a porous layer based on a viscous pore closure model (see Materials and Methods) (*38*), we obtain the curves shown in Fig. 7 (C and D). In this case, our estimated north polar heat flux aligns even better with the values implied by the ice shell thickness estimates in (*8*–*10*), while the disagreement with (*23*) becomes worse (Fig. 7C). The porosity values adopted for Fig. 7 (C and D) were not based on attempting to fit the observations—we simply used the example porosity structure in (*38*). If we use the shape model in (*23*) to extrapolate (Materials and Methods) our result to the global mean thickness (dotted gray rectangle in Fig. 7D), we obtain a result that is inconsistent with the global mean estimated in (*23*) (solid purple rectangle in Fig. 7D). Assuming still greater porosity would push our results even further out of alignment with (*23*). This suggests that if the shape model in (*23*) is accurate, we would require a different set of assumptions (e.g., allowing for internal heating or higher thermal conductivity) for our estimated north polar heat flux to be consistent with the ice shell structure in (*23*).

## DISCUSSION

The low effective emissivity for the north polar stare is unexpected (*27*, *39*), and temperature dependence has not been previously identified in observations. No emissivity features were found in (*27*), although the study focused on observations of warm (>70 K) scenes, and so the presence of emissivity features in observations at lower temperatures is not precluded.

A variety of physical mechanisms could contribute to a temperature-dependent ε′ on Enceladus. For the coldest scenes (<50 K), constituents other than water could be deposited at the north pole. These include seasonal CO_2_ “frost” and persistent CO_2_ hydrates [clathrates, (*40*)]; the former would have thermal properties that resemble amorphous water ice, and the latter has a lower emissivity than water ice.

A temperature-related effective emissivity could arise from temperature-dependent changes to both its real (*41*) and imaginary (*28*) refractive indexes, its grain size variations, and the properties of water ice (e.g., heat capacity). For example, models show that for wavelengths relevant for CIRS FP1, the change in the imaginary part of the refractive index of water ice results in decreased hemispherical emissivity at wave numbers below 70 cm^−1^ and above 120 cm^−1^ (*28*, *42*). Enceladus’ shell is almost purely water ice (uniquely in Saturn’s icy moons), so any implications from the fundamental properties of water ice should be more readily apparent here.

Spectral emissivity is dependent upon ice grain size. Spectral and hemispheric emissivities have been modeled for a variety of grain sizes identified on Enceladus (*42*, *43*). Because of lower spectral emissivities at the lowest wave numbers for some ice grain sizes (*28*, *42*), a resultant temperature dependence of ε′ would be made more apparent at lower (<50 K) temperatures. To illustrate this effect, we take two examples that specifically relate to the winter (33 K) and summer observations (70 K). If a 64 (256)–μm water ice grain emissivity spectrum [from figure 6a in (*42*)] is convolved with a Planck function at 33 K and a temperature and ε′ is fit to the resultant spectrum without and (with) our fitting approach (see Materials and Methods), we obtain a gray body temperature of 39 (35) K (2 s.f.) and ε′ of 0.37 (0.67). These numbers are not dissimilar to the relative blackbody and gray body fits to the CIRS north polar winter stare spectrum (38 K and 0.58). At 70 K, the same convolution results in a fit temperature of 74.5 (70.5) K and ε′ of 0.65 (0.8). Figure 6 shows that the range of summer polar temperatures is 70 to 72 K, fit as a blackbody. When fit as a temperature with joint emissivity, a temperature range of 71 to 77 K, with ε′ from 0.81 to 0.99, is found. At lower temperatures, the relative total emittance is disproportionately reduced compared to a Planck function peaking at higher wave numbers. This is a limited model that reproduces the tendency of the observed temperature-dependent ε′. Both emissivity spectra feature the roll-off in water ice emissivity at low wave numbers described in (*28*) and imply that emissivity becomes a more important consideration at the lowest scene temperatures. Emissivity relating to water ice grain size is explored with more complexity in (*28*, *44*), but these studies did not consider the lowest (<50 K) temperatures found on Enceladus. Emissivity in the spectral range of FP1 should be a consideration for future thermal surface and microphysical modeling of Enceladus’s colder surfaces. Further laboratory measurements of the conductive and radiative properties of water ice grains at temperatures of 20 to 50 K would aid in the analysis of thermal remote sensing observations and improve current thermal microphysical models.

If we ignore any impacts of temperature-dependent ε′ in the model (implemented to more accurately model the thermal history and emittance of the surface), the estimate of endogenic power slightly increases. If the north polar stare observation is modeled as a blackbody using the expected thermal inertia and albedo (Materials and Methods), then the predicted passive temperature is 23.6 K (17.6 mW m^−2^ emitted power). The blackbody fit to the CIRS spectrum is 33 K, equivalent to 67.2 mW m^−2^, a difference of 49.6 mW m^−2^. If we take the blackbody model result and instantaneously imposed the observed emissivity (although incongruent with the model’s thermal history), the excess endogenic power would be 51.2 mW m^−2^. The gray body fit to the observation (38 K and ε′ of 0.58) has a difference if 50.1 mW m^−2^ above the passive model expected emission.

As shown in Fig. 1A, there are no FOVs with a temperature lower than 30 K, meaning that if the observed endogenic heat results from hotspots within a passive background, this cannot be readily decomposed with the FOV of this size. This also does not preclude the presence of hotspots in the north polar region in addition to general conductive heat flow. Defining the true nature of the heat distribution at the north pole will require new observations in north polar winter with a smaller FOV yet the same (or improved) sensitivity to detect the very low temperatures of CIRS FP1.

Assuming a conductive ice shell, our estimated heat flux in the north polar region implies an ice shell thickness of 20 to 23 km (global mean of 25 to 28 km), which falls within the range of values estimated by several previous ice shell structure models on the basis of the estimated shape, gravity, and rotation state of Enceladus (*8*–*10*, *23*)—a set of measurements that is entirely independent of ours. Adding a porous ice layer reduces the implied shell thickness, improving the alignment between our implied north polar ice shell thickness and that of a subset of the published ice shell structure models (*8*–*10*). Specifically, with our porosity assumptions (see Materials and Methods), we estimate a north polar shell thickness of 16 to 18 km (global mean of 21 to 23 km), in good agreement with (*8*–*10*). Meanwhile, if we adopt the ice shell structure and shape model in (*23*), we are unable to obtain internally consistent results with a purely conductive ice shell. This analysis highlights the importance of resolving discrepancies between existing shape models for estimating the global conductive heat flux and obtaining thermal information on multiple timescales to constrain conductive heat flow. While Europa’s seasonality is less extreme than Enceladus’, it remains vital that upcoming missions to Europa (Europa Clipper and Juice) measure its thermal emission at multiple local times to best enable similar analysis.

We have argued above that the most likely explanation for the north polar CIRS observations is the presence of 46 ± 4 mW m^−2^ endogenic heat derived from conductive heat flow through the ice shell, which is relatively thin at the poles. This implies a north polar power output estimate of 1.7 GW (to 65°N). Given that the rest of Enceladus (except the SPT) probably has a thicker shell, an upper bound on non-SPT heat loss is 35 GW (north of 65°S). [We can refine this limit further, assuming the shape models in Fig. 7 (B and D).] The observation-derived upper limit of 19 GW of endogenic heat loss from the SPT (*18*, *19*) means that the total heat loss from Enceladus should not exceed 54 GW. This heat loss estimate may then be compared with estimates of tidal heat production, which are in turn based on astrometric measurements (*1*). For example, ref. (*25*) reports the best model estimates of current equilibrium tidal heating rates to be 50 to 55 GW (but with uncertainty ranges of more than 100%). The similarity of the estimated heating and heat loss rates suggests the ocean in its current epoch is long-lived, making it far more likely to be an environment hospitable to the development of life (*2*).

## MATERIALS AND METHODS

### CIRS observations

Cassini’s CIRS was the principal instrument used to provide accurate observations of Enceladus’ surface temperature, detecting infrared light between 10 and 1400 cm^−1^ (7 to 1000 μm). FP1 covered 10 to 600 cm^−1^ (16.7 to 1000 μm) and had a single circular detector (3.9 mrad) with an approximately Gaussian detector response within the FOV (*45*). This means that the FOV has maximum sensitivity toward the center (Fig. 4A). CIRS Focal Plane 3 (600 to 1100 cm^−1^, 9.1 to 16.7 μm) had 10 detectors and higher spatial resolution (0.273 mrad), but its shorter wavelength coverage and higher detector temperature made it insensitive to the colder scenes <80 K outside the SPT.

Stare observations were an opportunity to generate averaged observations where the noise would be much reduced to enhance their quality. This is particularly useful for very cold scenes (<50 K), which have a low signal-to-noise ratio. The averaged spectrum made on 14 July 2005 shown in Fig. 1 comprises 110 individual sequential measurements made over 518 s. The spectral resolution of the original interferometer spectra used is 5 cm^−1^. The onboard clock spacecraft event time range is 1121373444 to 1121372926, and the subsolar latitude is 21.08°N. The averaged FOV has an area of 5450 km^2^, with ellipse axes of 79.1 and 96.5 km. The lowest latitude of the FOV is 77.4°N. A similar subset of measurements was used in (*17*) as a contemporaneous contrast to those of the substantially hotter sulci at the south pole when they were first identified.

The summer FP1 observations shown in Fig. 6 were made on 14 October 2015. The spacecraft event time ranges from 1444818552 to 1444818845 and ranges in latitude from 67°N to 87°N. Their areas range between 560 and 1480 km^2^, with semimajor/minor axes ranging from 33/21 to 58/33 km, respectively. The emission angles range from 50° to 72° with phase angles of <10°. The summer CIRS spectra are well fit by a single temperature Planck function as shown in the Supplementary Materials (figs. S1 to S4).

### Fitting of temperature and error

CIRS FP1 captures the bulk of thermal emission of Enceladus’ surface temperature range and, as such, may be fit by a Planck function for the determination of temperature. This methodology is discussed exhaustively in (*29*, *33*, *39*) and described briefly here. A blackbody curve is fit to the observed spectrum using chi-squared minimization within the routine BBCUBEFIT written in IDL. It can also jointly fit a blackbody temperature and effective emissivity (ε′) value. Temperature uncertainty estimates are based on the standard deviation of the fit residual for each measurement. They are used to produce a random synthetic noise spectrum of the equivalent magnitude, which is added to the fitted Planck function. We use a fit window of 30 to 275 cm^−1^. This synthetic spectrum is subsequently fit to yield a temperature that is equally representative of the original fit to the observation and then repeated (100 times) to generate a distribution of fit temperatures. The standard deviation of this distribution produces a symmetrical error bound centered around the fit temperature. An improvement in the determination of errors for cold scenes is highlighted in (*33*) in terms of establishing asymmetric errors for scenes that have the highest noise, because the distributions themselves become highly skewed. This makes the lower uncertainty bound larger but enables more appropriate (smaller) uncertainty to be expressed in the upper bound (*29*, *46*). Our method of estimating the temperature error does not necessarily indicate whether the assumed fit paradigm (blackbody, gray body, or more than one temperature within the FOV) is appropriate. This can only be ascertained by inspection of the residual or the fit itself.

### Global emissivity study

Where ε′ < 1, BBCUBEFIT can jointly fit temperature and ε′ and similarly produces error estimates for both parameters. Effective emissivity derived from CIRS FP1 spectra has uncertainties associated with them commensurate with the increasing noise quotient at lower radiances when they are derived from observations of cold scenes, which we define as <50 K. In such scenes, the relative emissivity error for a single FOV is typically >20%. The noise is reduced by the averaging of the stare observation, which confers a lower error on ε′ as well as temperature. As such, accurate global mapping of ε′ is much improved by averaging spectra where possible. We identified a set of “quasistares,” each with a minimum of 10 spectra from overlapping or contiguous observations taken anywhere on Enceladus north of 50°S (to exclude the effects of SPT). The quasistares are defined as having the centers of the FOV within 8 km of each other (equivalent to 2° latitude and maximum insolation change of <4%), taken within 100 s (to minimize local time differences). Individual FOVs are only included in one averaged spectrum, and spectra are either completely insolated or not, with observations over the terminator excluded to minimize temperature variation within the FOV. We found that averaging these spectra typically reduces the error associated with the emissivity fit by between half and a factor of 10.

To account for this changing ε′, we derive a first-order polynomial fit to the data from quasistares outside the SPT (the green line in Fig. 3B), which gives the following empirical relationship

$$\varepsilon^{\prime }=0.0094T+0.2612$$ (1)

We implemented this temperature and ε′ relationship directly into the thermprojrs surface thermal model where emissivity is updated iteratively with model temperature at each time step. This results in a surface that radiates efficiently in summer and less in winter, yielding a higher winter surface gray body temperature. Not accounting for this temperature-dependent seasonal change in surface emissivity would lead to an underestimation of model temperature in winter and therefore overestimation of any apparent endogenic heat inferred.

### Modeling surface temperature

The surface temperatures of Enceladus and other icy moons in the Saturn system have been extensively modeled in (*29*, *39*), using the one-dimensional surface temperature model thermprojrs (*46*), which has been shown to reproduce expected temperatures well (*47*). For polar observations, we require the seasonal version of the model as described elsewhere (*29*, *33*). This model uses bulk thermal conductivity, thermal inertia, bolometric albedo, and emissivity as input thermal parameters. The global average thermal inertia ( $15_{-9}^{+24}$ MKS) and albedo (0.81 ± 0.04) for Enceladus are reported in (*30*), derived using CIRS FP1 data. To model the spectrum as a blackbody, unit emissivity was used and thermprojrs was updated to optionally use the temperature-dependent emissivity derived in this work. The most northern values given in (*11*) are $16_{-13}^{+17}$ MKS and 0.76 ± 0.6, respectively, for 60°N to 70°N. Estimates of bolometric albedo have also been derived from VIMS (*12*). They report values distinguishing between the leading (0.77 ± 0.09) and trailing hemispheres (0.93 ± 0.11) and suggesting a global average (0.85 ± 0.11), all of which are higher than the infrared-derived bolometric albedos. A global average bolometric Bond albedo of 0.76 ± 0.03 was calculated using observations from the Hubble Space Telescope as well as Cassini’s VIMS, Imaging Science Subsystem, and Ultraviolet Imaging Spectrograph (*13*), spanning a wider wavelength window than those in (*11*) or (*12*). This range of values highlights why the sensitivity of the modeled surface temperature to these variables requires evaluation to estimate their effect on the conferred temperature to represent model uncertainty.

The surface of Enceladus is predominantly water ice, so for the baseline passive model temperature, we use a specific heat of 0.8 J K^−1^ g^−1^ [for H_2_O (*48*)] and a density of 900 kg m^−3^. Because of the location of the stare, we use the northern-most values for thermal inertia and its uncertainty of 16 MKS and bolometric albedo/uncertainty of 0.76 (*11*) and the expected albedo value equal to the global average found in (*13*). This assumption is supported by panchromatic images (maps) that do not show a notable discontinuity in albedo toward the poles (either brighter or darker). The model temperature is determined at latitude and longitude intervals of 5° and 20°, respectively, which are appropriate for the extent of the spatial domain of the FOV over the pole. The model has a temporal sampling of 100 time steps per day.

The detector response projected onto the FOV (Fig. 4A) is interpolated onto the model grid. Using specified emissivity, each temperature is converted into a Planck radiance and weighted by the detector response at that point. It is then combined to form a forward modeled radiance that may be fit by BBCUBEFIT and can be directly compared to the observed spectrum.

### Roughness and sub-FOV temperature variation

The sub-FOV temperature variation at Rhea’s winter south pole was evaluated in (*33*), explicitly modeling surface facets using a digital elevation model using an adapted version of thermprojrs (also used in the present study) to determine whether sub-FOV temperature modeling was required to adequately fit the observation. The study found that when a realistic representation of roughness is included, the temperature variation within the scene increases. However, toward the winter pole (south of 80°S), the temperature variations in the rough thermal model were smaller than 1 K [figure 5 in (*33*)]—because of Rhea having been in winter darkness for several years.

Enceladus has an overall smoother and younger surface than Rhea, which is further from Saturn than Enceladus, making it less influenced by Saturn shine. To confirm this result for Enceladus, we implemented the same model for its north polar region where a thermal dichotomy is evident between the Saturn-facing and anti-Saturn hemispheres (Fig. 4). We assume an extreme facet slope of 50° on Saturn-facing and anti-Saturn hemispheres at all azimuths. Figure 4A shows that detector sensitivity is dominated by emission from 85°N and northward. Using an endogenic heat flow up to 46 mW m^−2^ with empirical temperature-dependent effective emissivity, the maximum temperature range (minimum to maximum) across the FOV are 4.1, 2.9, and 2.6 K at latitudes of 82°N, 85°N, and 87°N, respectively. The changes in temperature for the slopes over the full azimuthal range are ±0.2, ±0.1, and ±0.1 K, different from when the same points are modeled as flat.

The model does not include the effects of shadow or self-heating from other facets. Considering a location close to the pole after a decade of no insolation and approaching near thermal equilibrium, we expect this to have a negligible impact in this case. Further model details are given in (*33*).

### Ice shell thickness and conducted heat

The relationship between ice shell thickness and conducted heat flow based on a temperature difference at the water/ice boundary and the surface when the thermal conductivity is temperature-dependent (*25*) is given by

$$F_{\text{cond}}=\left(1-\frac{d}{R}\right)\frac{c}{d}\text{ln}\left(\frac{T_{m}}{T_{s}}\right)$$ (2)

Here, *F*_cond_ is the conductive heat flow, *c* is a constant (651 W/m) [(*49*), p. 43], *d* is the thickness of the ice shell, *T*_m_ is the temperature at the base of the shell, and *T*_s_ is the annual mean surface temperature. The term in the first parentheses is required to account for the spherical geometry, the effect of which is nonnegligible for a body as small as Enceladus. We take *T*_m_ = 270 K. Equation 2 assumes that no heat dissipation occurs in the shell itself. Estimating the precise regional mean annual surface temperature is challenging but affects estimates of ice shell thickness (or similarly conducted heat based on a model ice thickness). We estimate the area weighted mean annual temperature using the passive model thermprojrs and the expected thermal inertia, albedo, and heat flow from (*11*) (specified above) to predict mean annual temperatures of 51 K north of 75°N and 60 K globally. Using this value and Eq. 2, we arrive at the ranges shown in Fig. 7.

### Shell thickness assuming isostatic compensation

Assuming Airy compensation under the “equal pressures” isostasy model in (*50*), the shell thickness, d(θ,ϕ) , is related to the nonhydrostatic (nh) topography, $h^{\text{nh}}\left(\theta ,\phi \right)$ , where θ,ϕ denote the latitude and longitude and the mean shell thickness, ${\underline{d}}_{\text{ice}}$ , by

$$d\left(\theta ,\phi \right)={\underline{d}}_{\text{ice}}+h^{\text{nh}}\left(\theta ,\phi \right)\left(1+\frac{\rho_{\text{ice}}}{\Delta \rho }\frac{g_{t}}{g_{b}}\right)$$ (3)

where $g_{t}$ and $g_{b}$ are, respectively, the gravity at the surface and at the base of the ice shell, and Δρ is the density contrast between the ice and the ocean. If we assume ice and ocean densities of 925 and 1020 kg/m^3^, respectively, and a mean ice shell thickness of 20 to 30 km, then the quantity in parentheses is roughly 10, meaning that every kilometer of nonhydrostatic topography translates to roughly 10 km of the additional shell thickness compared to the mean. Given that the north pole lies ~500 m below the reference equipotential surface [assuming the shape model in (*34*)], this means that we can expect the north polar ice shell thickness to be roughly 5 km less than the global mean shell thickness. Hence, given that our north polar heat flux estimate suggests a north polar shell thickness of 20 to 23 km (solid black rectangle in Fig. 7A), this polar thickness corresponds to a global mean ice shell thickness of 25 to 28 km (dotted black rectangle in Fig. 7B), which lies somewhere between 19 and 24 km estimated in (*10*) and 27 and 33 km estimated in (*23*). If we instead follow the “equal weights” isostasy model (*9*), the factor in parentheses is closer to 12, making the offset between the global mean and the north polar topography ~20% larger [in Fig. 7, we used this for the north polar extrapolation of the result in (*9*) only; for the rest, we use Eq. 3]. Note that ref. (*9*) did not report uncertainties; we arbitrarily assigned an uncertainty of ±2.5 km [roughly comparable to the uncertainties reported in (*10*)] to their result to facilitate comparison with the other results shown in Fig. 7. Results are also sensitive to the assumed shape model. For example, whereas the shape model in (*35*) [adopted in (*8*)] and the shape model in (*34*) [adopted in (*9*, *10*)] exhibit a topographic depression at the north pole (relative to an equipotential surface), the north polar region in the shape model in (*23*) stands ~100 m above the reference equipotential. This means that the north polar thickness would be roughly 1 km greater than the global mean. Hence, if we adopt the shape model in (*23*), our inferred north polar shell thickness of 20 to 23 km would instead imply a global mean thickness of 18 to 22 km (dotted gray rectangle in Fig. 7B).

We consider the effect of porosity in Fig. 7 (C and D). The heat flux is reduced by a factor of 1 + (*g*[1 − *f*]/*f*), where *g* is the fraction of the shell thickness that is porous, and *f* is the factor by which the porous layer is lower in conductivity. On the basis of the results of figure 1 in (*38*), we take specimen values of *g* = 0.3 and *f* = 0.5, so the heat flux is reduced by a factor of 1.3. The overall effect is to imply a thinner ice shell for a given conductive heat flow.
