# Supplementary material for: Endogenic heat at Enceladus’ north pole
Source: Sci Adv. 2025 Nov 7;11(45):eadx4338. doi: 10.1126/sciadv.adx4338 (PMC12594170; doi:10.1126/sciadv.adx4338)
Supplement: Supplementary file 1 — Figs. S1 to S4 [file sciadv.adx4338_sm.pdf]

Science Advances
AAAS

Supplementary Materials for

Endogenic heat at Enceladus' north pole

Georgina Miles *et al.*

Corresponding author: Carly J. A. Howett, carly.howett@physics.ox.ac.uk

Sci. Adv. **11**, eadx4338 (2025)
DOI: 10.1126/sciadv.adx4338

This PDF file includes:

Figs. S1 to S4

Supplementary Text

Data Description

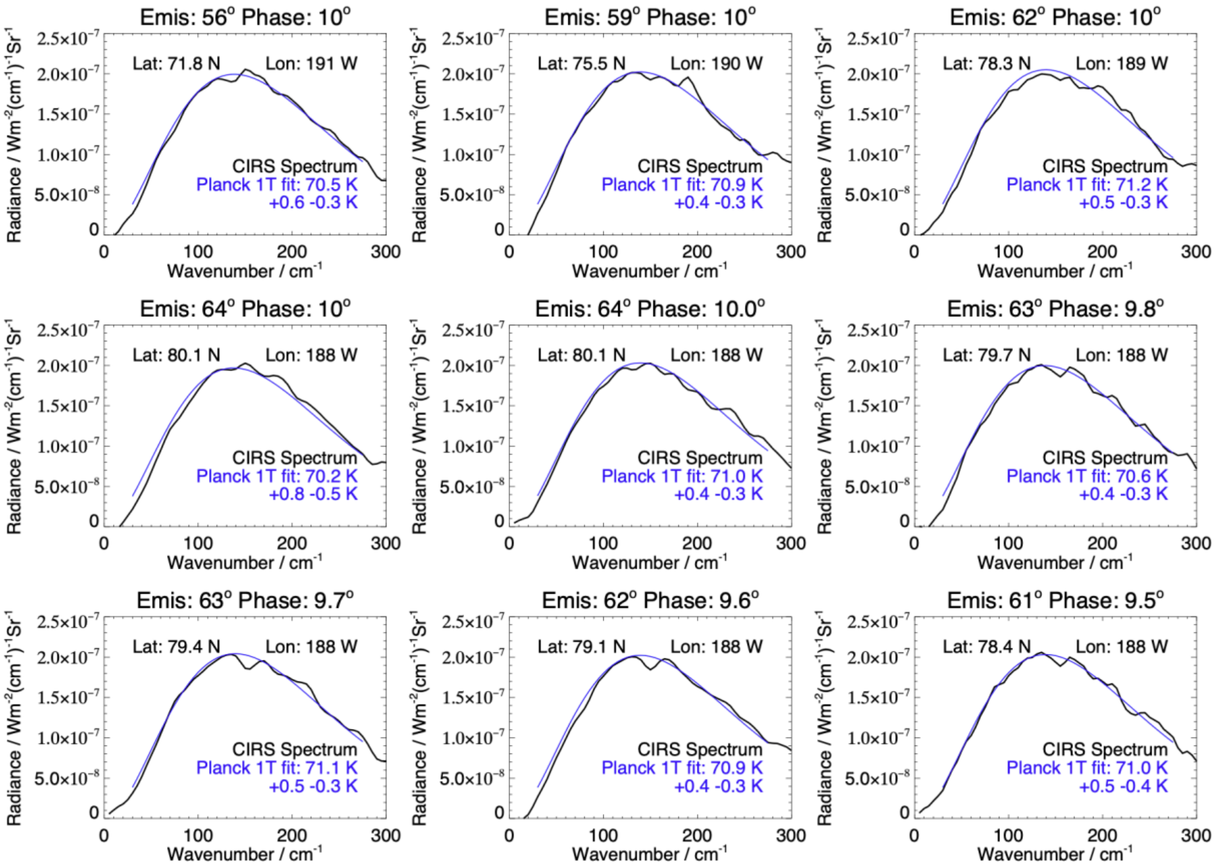

Fig. S1. Summer FP1 observations shown in Fig. 6 made on 14th October 2015.

CIRS FP1 observation from top left to bottom right, SCET (onboard clock) values:1444818552, 1444818557, 1444818562, 1444818566, 1444818571, 1444818581, 1444818586, 1444818591, 1444818596. Emission and phase angle stated above each panel. CIRS spectrum is shown in black, fitted blackbody and temperature with fit errors shown in blue. See manuscript text for further information.

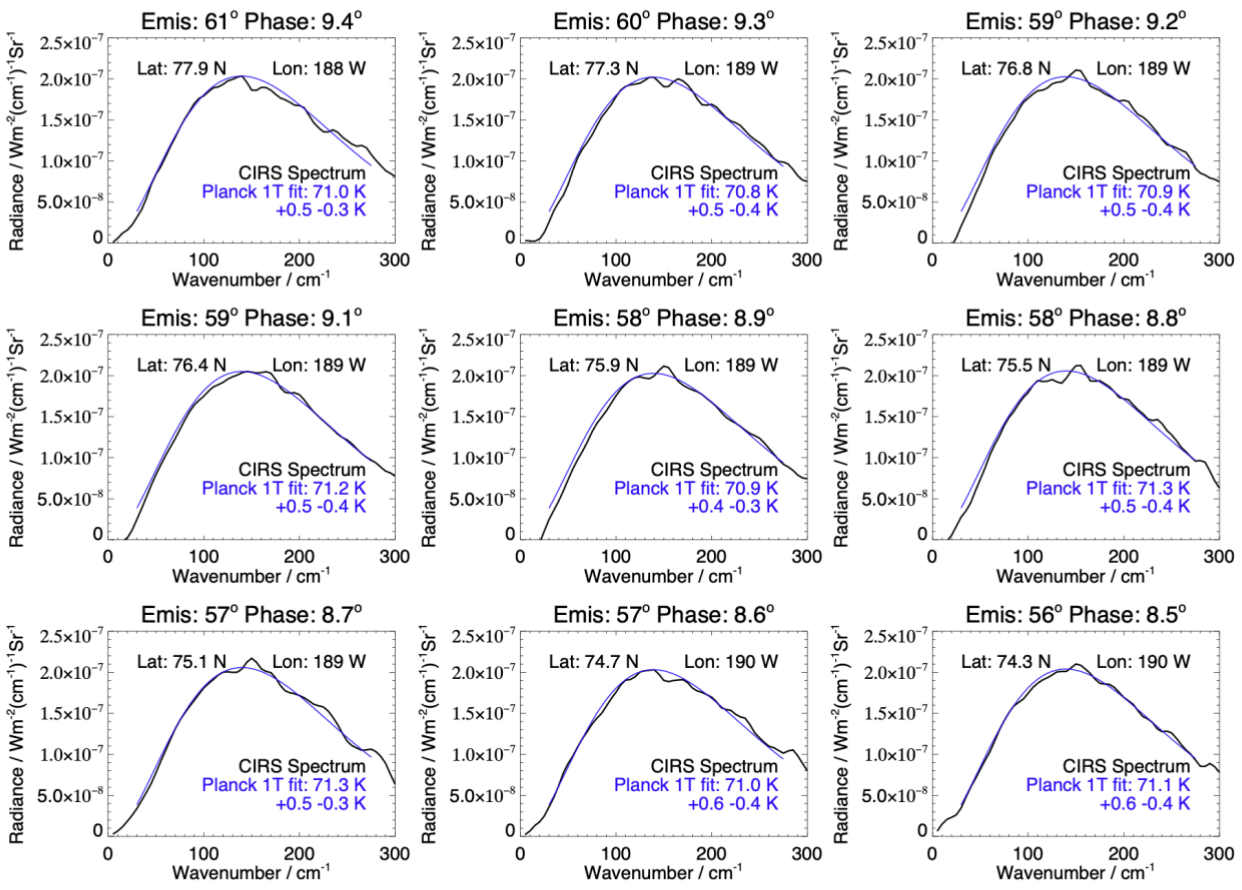

Fig. S2. Summer FP1 observations shown in Fig. 6 made on 14th October 2015.

CIRS FP1 observation from top left to bottom right, SCET (onboard clock) values:1444818601, 1444818606, 1444818610, 1444818615, 1444818620, 1444818625, 1444818630, 1444818635, 1444818640. Emission and phase angle stated above each panel. CIRS spectrum is shown in black, fitted blackbody and temperature with fit errors shown in blue. See manuscript text for further information.

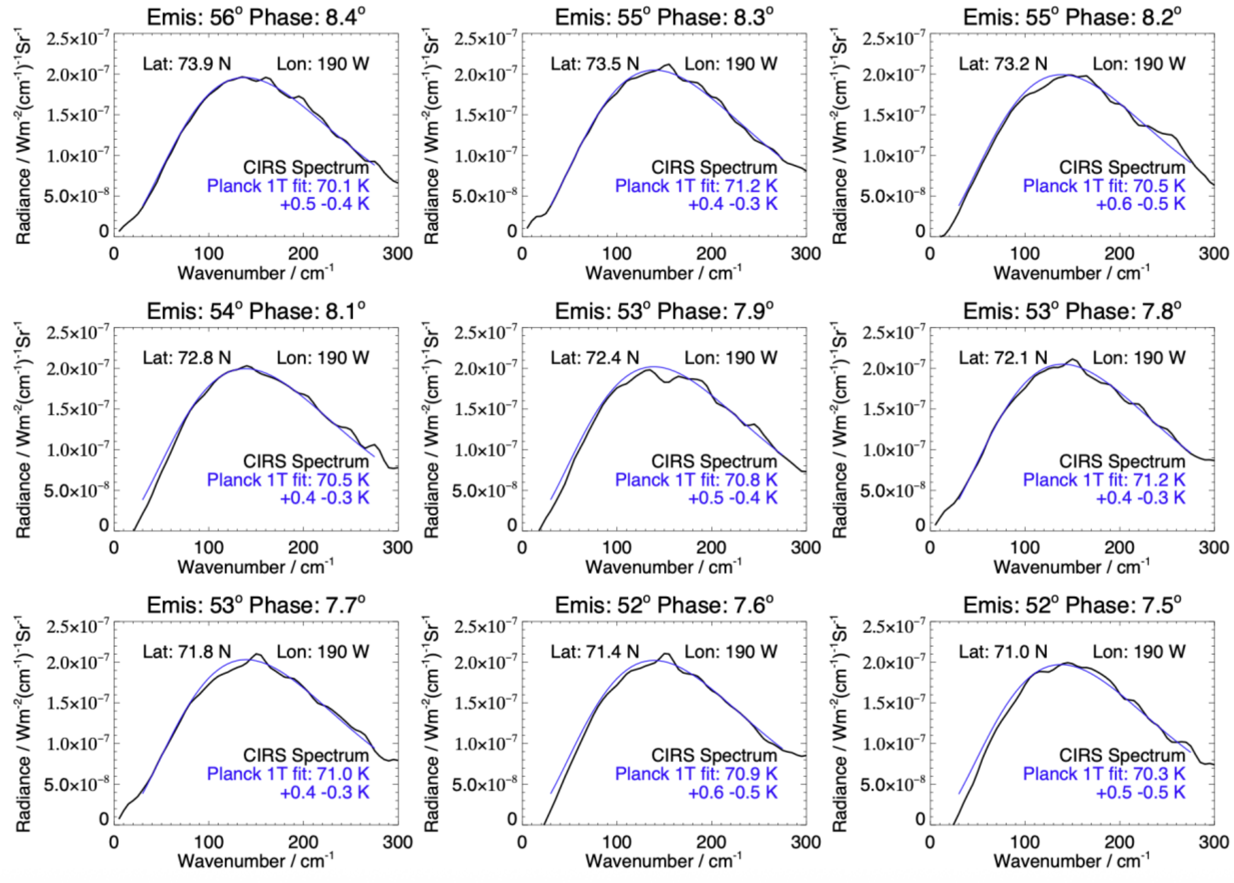

Fig. S3. Summer FP1 observations shown in Fig. 6 made on 14th October 2015.

CIRS FP1 observation from top left to bottom right, SCET (onboard clock) values:1444818645, 1444818649, 1444818654, 1444818659, 1444818664, 1444818669, 1444818674, 1444818679, 1444818684. Emission and phase angle stated above each panel. CIRS spectrum is shown in black, fitted blackbody and temperature with fit errors shown in blue. See manuscript text for further information.

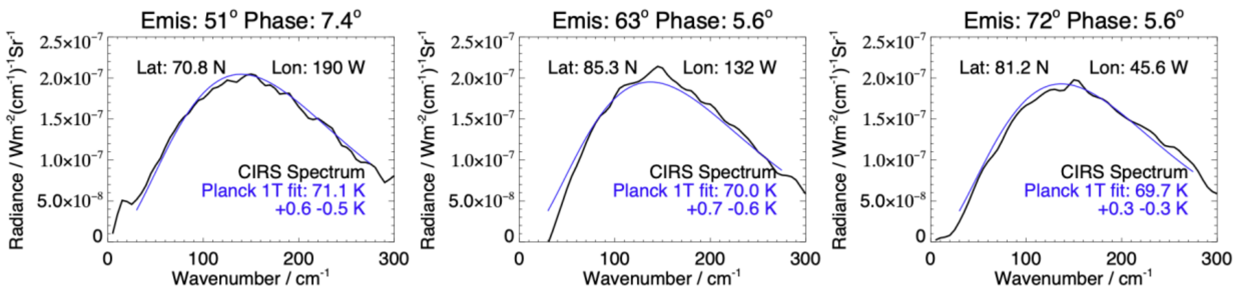

Fig. S4. Summer FP1 observations shown in Fig. 6 made on 14th October 2015

CIRS FP1 observation from top left to bottom right, SCET (onboard clock) values: 1444818688, 1444818840, 1444818845. Emission and phase angle stated above each panel. CIRS spectrum is shown in black, fitted blackbody and temperature with fit errors shown in blue. See manuscript text for further information.
